# Low-Frequency Pulsed Magnetic Field Improves Depression-Like Behaviors and Cognitive Impairments in Depressive Rats Mainly via Modulating Synaptic Function

**DOI:** 10.3389/fnins.2019.00820

**Published:** 2019-08-20

**Authors:** Jiajia Yang, Ling Wang, Faqi Wang, Xiaoxuan Tang, Peng Zhou, Rong Liang, Chenguang Zheng, Dong Ming

**Affiliations:** ^1^Laboratory of Neural Engineering and Rehabilitation, Department of Biomedical Engineering, College of Precision Instruments and Optoelectronics Engineering, Tianjin University, Tianjin, China; ^2^Tianjin International Joint Research Center for Neural Engineering, Academy of Medical Engineering and Translational Medicine, Tianjin University, Tianjin, China

**Keywords:** depression, low-frequency pulsed magnetic field, cognition function, synaptic plasticity, neuronal oscillation

## Abstract

Transcranial magnetic stimulation (TMS) has shown great promise as a medical treatment of depression. The effectiveness of TMS treatment at high frequency has been well investigated; however, low-frequency TMS in depression treatment has rarely been investigated in depression-induced cognitive deficits. Herein, this study was carried out to assess the possible modulatory role of low-frequency pulsed magnetic field (LFPMF) on reversing cognitive impairment in a model of depression induced by chronic unpredictable stress (CUS). Wistar rats were randomly allocated into four groups as follows: a control group (CON), a control applied with LFPMF (CON + LFPMF), a CUS group, and a CUS treated with LFPMF (CUS + LFPMF) group. During 8 weeks of CUS, compared to those in the CON group, animals not only gained less weight but also exhibited anhedonia, anxiety, and cognitive decline in behavioral tests. After 2-week treatment of LFPMF, a 20 mT, 1 Hz magnetic stimulation, it reversed the impairment of spatial cognition as well as hippocampal synaptic function including long-term potentiation and related protein expression. Thus, LFPMF has shown effectively improvements on depressant behavior and cognitive dysfunction in CUS rats, possibly via regulating synaptic function.

## Introduction

Depression is a major neuropsychological disorder and has the third-largest disease burden, with around 350 million patients suffering from it globally as of 2012 (Smith, 2014). It is a chronic disease and exhibits a wide variety of symptoms, such as depressive mood, sluggish ideation, and suicidal ideation (Pazini et al., 2016). Depressive disorder may lead to burden of patients, their families, and society. Besides emotional problems, recently researchers have frequently observed depressed patients with cognitive impairment (Lam et al., 2014; Rock et al., 2014; Trivedi and Greer, 2014). Furthermore, one third to half of depressed patients who are in remission are still suffering from the cognitive deficits (Rosenblat et al., 2016). Although understanding of the pathophysiology of depression is still rudimentary due to its complex etiology. Previous findings suggest that neuronal activity, neural plasticity, oxidative stress, and cortisol levels contribute to the pathogenesis of depression (Neylan et al., 2001; Bremner et al., 2004; Hassouna et al., 2016). Among them, neural plasticity and oxidative stress also have great impact on cognition. And many antidepressants are beneficial to cognitive function in depression, such as vortioxetine and duloxetine (McIntyre and Lee, 2016). Cognitive dysfunction refers to significant and persistent functional impairment, which has attracted increased attention in the treatment of depression.

Transcranial magnetic stimulation (TMS), as a non-invasive brain stimulation technique, modulates brain activity via electromagnetic pulses discharged through a coil placed over the subject’s head (Myczkowski et al., 2018). This technology has been used for the treatment of many neurological and psychiatric disorders (Sachdev et al., 2002). Furthermore, repetitive transcranial magnetic stimulation (rTMS) has been approved by the United States FDA for medication-resistant depression (O’Reardon et al., 2010). At present, the majority of studies have focused on the effects of high-frequency TMS (>1 Hz) on depression (Lage et al., 2016), at which the cognition is indeed improved in depressive patients (Galletly et al., 2016; Myczkowski et al., 2018). However, the potential mechanism has not been clarified yet.

Recently, Huang et al. (2017) reported that low-frequency rTMS (1 Hz) treatment alleviated the deficits of AD-related cognitive function and synaptic plasticity. These results caused concerns about the role of low-frequency (≤1 Hz) magnetic fields on brain function. According to previous investigations, low-frequency pulsed magnetic field (LFPMF) could regulate synaptic functions and cortical excitability (Chauviere et al., 2009; Guosheng et al., 2014; Senkowski and Gallinat, 2015), which suggested that LFPMF may play a particular role on the neuromodulation of brain. Furthermore, according to a conductance-based neuron model, LFPMF could modulate neuronal activities, such as changing spike times and further modulating spiking rhythms (Guosheng et al., 2014). Because rhythmic or repetitive neural activity of neuronal ensembles, also called as neural oscillation, has been demonstrated to be closely associated with the cognitive functions (Winson, 1978; Chauviere et al., 2009; Ehrlichman et al., 2009; Senkowski and Gallinat, 2015), LFPMF may have the potential to be applied to reduce depression-induced cognition impairment.

Herein, in the present study we recorded local field potential (LFP) and excitatory postsynaptic potential (EPSP) in hippocampal CA1 and CA3 regions to explore the alternation of synaptic plasticity and neural oscillations. Meanwhile, western blot assay was performed for measuring synaptic-associated protein alternations to explore the potential synaptic mechanism. Overall, we indicate LFPMF (1 Hz) can ameliorate depression-like behaviors and cognitive impairment in a model of depression induced by chronic unpredictable stress (CUS), as well as the potential mechanisms.

## Materials and Methods

### Animals

**FIGURE 1:**
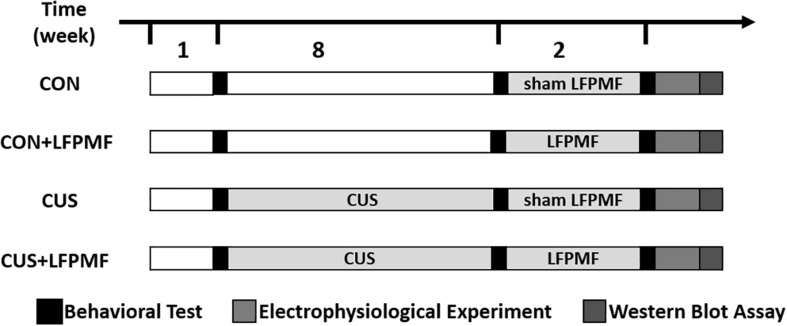
Experimental protocol.

Thirty-six adult male Wistar rats were purchased from the Laboratory Animal Center, Academy of Military Medical Science of People’s Liberation Army, and they were allowed to habituate for 1 week. Unless otherwise specified, animals were kept under a 12-h light-dark cycle and allowed *ad libitum* access to food and water. They were randomly divided into control group (*n* = 16) and model group (*n* = 20). The rats of the model group received the CUS procedure for 8 weeks, while control group rats were with standard housing. Unfortunately, one control group rat died of fighting and one of the model group died during the CUS procedure. After 8 weeks, rats of the model group were randomly divided into LFPMF (CUS + LFPMF, *n* = 10) or standard housing (CUS, *n* = 9). Control group rats were randomized into two groups, CON (*n* = 8, standard housing) and CON + LFPMF (*n* = 7, LFPMF). All experiments were performed in accordance with the Animal Management Rules of the Ministry of Health of the People’s Republic of China. All animal experiments were approved by the Animal Research Ethics Committee, School of Medicine, Nankai University. The experimental schedules are depicted in Figure 1.

### CUS Procedure

To induce chronic stress in rats from the model group, we used a previously validated CUS protocol with some modifications (Willner et al., 1987, 1992; Zhang et al., 2018). Rats were subjected to various and repeated unpredictable stressors for a period of 8 weeks. Stressors were from the following list: ice–water swim (4°C ± 2 for 5 min), reversal of dark/light cycle, white noise (60 min), hot-water swim (40°C ± 2 for 5 min), clamping tail (1 min), tilt cages (45° tilt), and food and water deprivation. Each rat received one stressor per day. In order to achieve unpredictability, the stressors were applied in a different sequence each week to avoid any habituation. At the same time, control rats did not receive any stressors and were housed in normal conditions.

### LFPMF Stimulation Procedure

During treatment, the round coil was placed on the head of awake animal at an approximately 5 mm distance from the skin. Each animal received 1 Hz pulsed trains at 20 mT magnetic field intensity, which were conducted during one LFPMF session in 14 days, 1 h per day. The rats were in the restraint device throughout their individual treatments.

### Behavioral Experiment

Sucrose preference test (SPT): prior to the SPT, all rats were trained to habituate 2% sucrose water by placing a bottle of normal water and a bottle of sucrose solution with them for 2 days. Afterward, they were deprived of food and water for 23 h. Then animals were exposed to one bottle of tap water and one bottle of 2% sucrose solution for 1 h. The sucrose preference index (SPI) is defined as the ratio of the sucrose consumption to the total amount of solution consumed (Yi-Huan et al., 2015). The test was performed before and after CUS procedure.

Anxiolytic activity was measured in the elevated plus-maze (EPM) test. In this test, rats were placed in a standard EPM sized maze. Animals were allowed to freely explore the maze for 5 min, and the total number of arm entries and the percentage of entries into and the proportion of time spent in the open arms were assessed (Hu et al., 2016). The test was performed after LFPMF or sham stimulation.

Morris water maze (MWM) was performed as described previously with some changes (Wang et al., 2017b). The whole task comprised four consecutive stages: acquisition training, probe trial, reversal training, and reversal probe trial. Training trials (days 1–4) consisted of eight sessions (two sessions per day, 7 h apart), each with four trials. One trial ended when the rat located the hidden platform. Rats were allowed to swim for a maximum of 60 s for each trial. At 24 h after the last training session, the rats were tested in the probe trial in which the platform was removed. In reversal phases, the platform was moved to the opposite quadrant. The test was performed after LFPMF or sham stimulation. All behaviors were carried out with the experimenter blind to the treatment groups.

### Electrophysiological Experiment

Animals were anaesthetized with urethane with a dosage of 4 ml/kg prior to placement in a stereotaxic frame. A monopolar extracellular stainless steel recording electrode was implanted into hippocampal CA1 region (2.5 mm lateral and 3.5 mm posterior to Bregma; depth from dura, 2.0–2.5 mm), while a concentric bipolar stainless steel electrode was placed into the Schaffer collaterals region (3.5 mm lateral and 4.2 mm posterior to Bregma; depth from dura, 2.5–3.0 mm). LFP signals were sampled simultaneously in both CA1 and Schaffer collaterals at a 1-kHz sample frequency. Before the long-term potentiation (LTP) induction, the test stimuli were delivered to CA1 region every minute to evoke a response of 70% of its maximum (range 0.3–0.5 mA). Afterward, theta burst stimulation (TBS) consisting of 30 bursts (12 pulses) of high-frequency stimulation (200 Hz) was used to induce LTP. The electrophysiological data were measured in Clampfit 10.0 (Molecular Devices, CA). More details were illustrated in the previous papers (Xu et al., 2015; Zheng and Zhang, 2015).

### LFP Analysis

All the LFP data processing was conducted offline using custom routines in MATLAB (MathWorks). In our paper, several mathematical methods were used to conduct hippocampal neurodynamic analysis, including power spectrum, sample entropy (SampEn), phase locking value (PLV), and modulation index (MI) of theta-gamma cross frequency coupling between CA1 and CA3 regions. The details were described in previous studies (Xu et al., 2015; Zheng and Zhang, 2015).

### Western Blot Assay

After electrophysiological experiment, the rats were sacrificed immediately and the following protocols reported previously with minor modification were conducted (Wang et al., 2017a; Yang et al., 2017). The hippocampus was removed at 0°C and homogenized in RIPA buffer which contained 1% PMSF (Solarbio, China). Lysates were then centrifuged at 12,000 rpm at 4°C for 20 min, and the supernatants were collected. Protein concentration was determined using the BCA Protein assay kit (Solarbio, China). After that, equal amounts of protein (40 ug/lane) for each sample were loaded and run on an 8–15% SDS-PAGE gel, which were transferred to 0.44 um polyvinylidene difluoride (PVDF) membrane (Millipore Corporation) at 4°C (BIO-RAD, United States). The PVDF membrane was blocked in Tris-buffered saline with Tween-20 (TBST) containing 5% skimmed milk for 1 h at room temperature. Next, the membranes were incubated with primary antibody overnight at 4°C (anti-SYP 1:10000, anti-PSD95, anti-NMDAR2B 1:2000, Genetex). After washing thrice with TBST, the PVDF membranes were subsequently incubated with secondary antibody (anti-mouse IgG HRP conjugate, anti-rabbit IgG HRP conjugate, 1:2000, Genetex) for 1 h at room temperature. Finally, a computerized chemiluminescent imaging system (Tanon Science & Technology, China) was employed to identify the protein band intensities.

### Statistical Analysis

Statistical analysis was carried out with SPSS 11.0. Results are expressed as the mean ± SEM. The data of weight, MWM and fEPSP slop were analyzed using three-way repeated measures ANOVA, with treatment × model as the between-subjects factor and measurement session as the within-subjects factor. All other data were evaluated by a two-way ANOVA with treatment × model as the between-subjects factor, followed by Bonferroni test as *post hoc* analysis for further examination of group differences. Significance level was set at *p* < 0.05.

## Results

### Representations of Anxiety-Related Behaviors

During the behavioral experiments, there were some accidents causing loss of data for some rats. Specifically, after the SPI test, two bottles of two CUS + LFPMF rats were found to be leaking. And during the EPM test, one rat in CON group and one in CON + LFPMF group always fell down from the elevated plus-maze. So during the analysis of behavioral data, there were CON (*n* = 7), CON + LFPMF (*n* = 6), CUS (*n* = 9), and CUS + LFPMF (*n* = 7).

**FIGURE 2:**
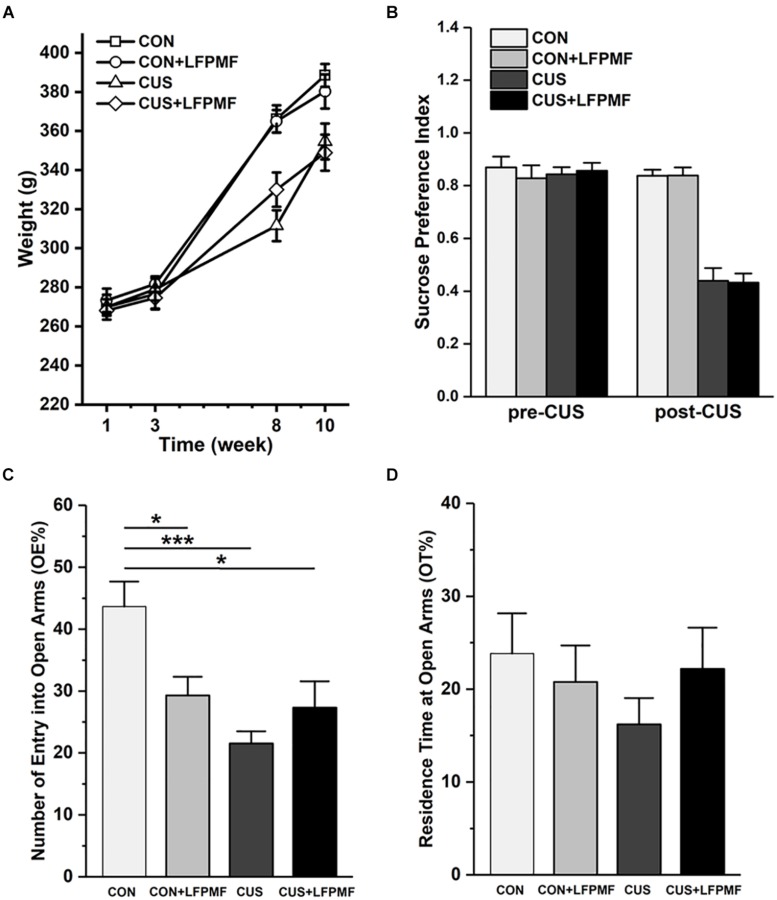
Effects of LFPMF and CUS on depressive- and anxiety-like behavior. **(A)** Mean weight. **(B)** Mean sucrose preference index. **(C)** Mean number of entries into open arms. **(D)** Mean residence time at open arms. ^∗^*p* < 0.05, ^∗∗∗^*p* < 0.001.

The effect of chronic stress on body weight is shown in Figure 2A. After 8 weeks, animal weight was significantly decreased in model group compared to the control group [main effect of CUS model: *F*(1,25) = 17.337, *p* < 0.001, repeated measures ANOVA]. However, the CUS rats’ weights were not increased after LFPMF treatment [main effect of treatment: *F*(1,25) = 0.021, *p* = 0.886, no CUS model × treatment interaction: *F*(1,25) = 0.035, *p* = 0.853, repeated measures ANOVA]. The results showed that after the CUS procedure, the SPI of the control group was significantly higher than that of the model group [Figure 2B, main effect of CUS model: *F*(1,25) = 108.153, *p* < 0.001, main effect of treatment: *F*(1,25) = 0.008, *p* = 0.929, no CUS model × treatment interaction: *F*(1,25) = 0.009, *p* = 0.925, two-way ANOVA], suggesting that CUS model rats behaved in anhedonia similarly as depressive patients. Analogously, in EPM compared to the control group, CUS-exposed rats exhibited anxiety behaviors as a decrease in the number percentage of entries into open arms [Figure 2C, main effect of CUS model: *F*(1,25) = 12.966, *p* = 0.001, main effect of treatment: *F*(1,25) = 1.628, *p* = 0.214, CUS model × treatment interaction: *F*(1,25) = 9.087, *p* = 0.006, two-way ANOVA] rather than the percentage of residence time in open arms [Figure 2D, main effect of CUS model: *F*(1,25) = 0.651, *p* = 0.427, main effect of treatment: *F*(1,25) = 0.141, *p* = 0.711, no CUS model × treatment interaction: *F*(1,25) = 1.369, *p* = 0.253, two-way ANOVA].

### LFPMF Prevents CUS-Induced Spatial Learning and Memory Deficits

**FIGURE 3:**
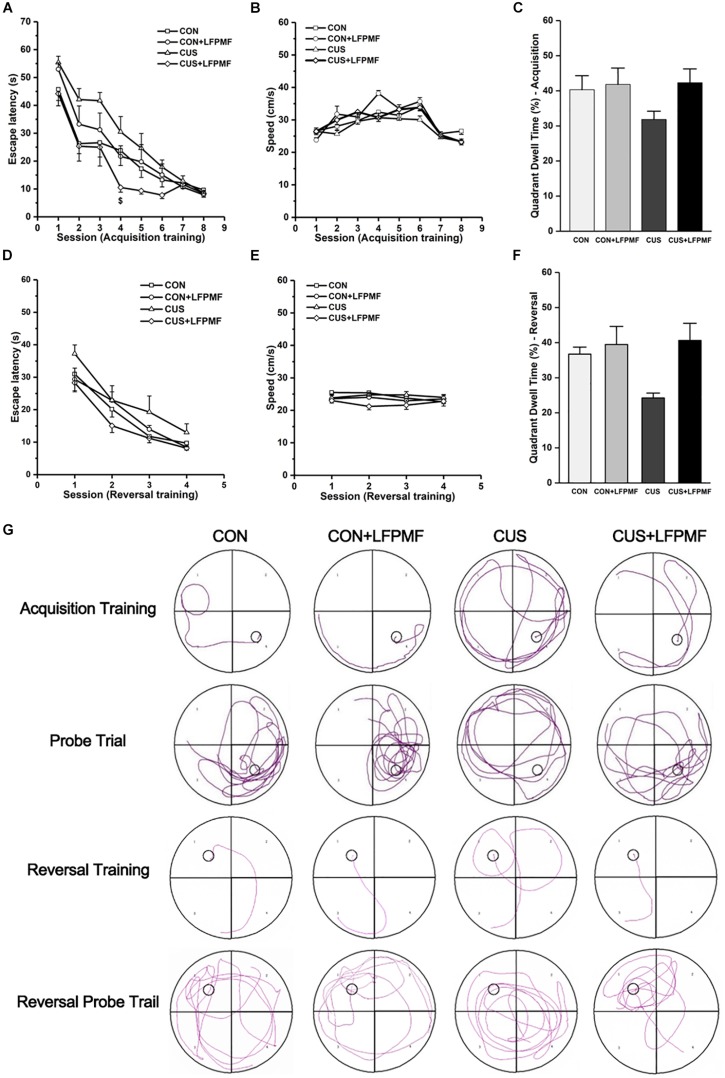
Effects of LFPMF on the improvement of spatial cognition. **(A,D)** Mean escape latency was calculated for each session in acquisition training and reversal training. **(B,E)** Mean swimming speed in acquisition training and reversal training. **(C,F)** Mean percentage of time spend in target quadrant in probe trial and reversal probe trial. **(G)** Representative swim tracks. ^$^CUS vs. CUS + LFPMF, *p* < 0.05.

Learning occurred in all groups as the escape latencies progressively became shorter over the training period [Figure 3A, main effect of time: *F*(7,175) = 66.327, *p* < 0.001; Figure 3D, main effect of time: *F*(3,75) = 52.087, *p* < 0.001, repeated measures ANOVA]. The rats with LFPMF treatment took less time to find the hidden platform than other groups in acquisition trainings of MWM test [main effect of treatment: *F*(1, 25) = 3.055, *p* = 0.093, main effect of CUS model: *F*(1, 25) = 0.043, *p* = 0.838, CUS model × treatment interaction: *F*(1,25) = 6.741, *p* = 0.016, repeated measures ANOVA]. Notably, this impairment could not be attributed to alterations of sensorimotor functions since the swimming speed remained unchanged among these groups (Figures 3B,E). After training, in the probe trial the rats showed robust spatial memory with a strong preference for the target quadrant [Figure 3C, main effect of treatment: *F*(1, 25) = 2.702, *p* = 0.113, main effect of CUS model: *F*(1, 25) = 1.185, *p* = 0.287, no CUS model × treatment interaction: *F*(1,25) = 1.503, *p* = 0.232, two-way ANOVA], as well as in the reversal probe trial [Figure 3F, main effect of treatment: *F*(1, 25) = 7.695, *p* = 0.010, main effect of CUS model: *F*(1, 25) = 2.714, *p* = 0.112, no CUS model × treatment interaction: *F*(1,25) = 3.922, *p* = 0.059, two-way ANOVA]. The results showed that LFPMF treatment impacted the reversal memory of rats. Besides the statistical results, Figure 3G shows representative training swim tracks in acquisition and reversal stages.

### The Effects of LFPMF on Synaptic Plasticity in Hippocampus

**FIGURE 4:**
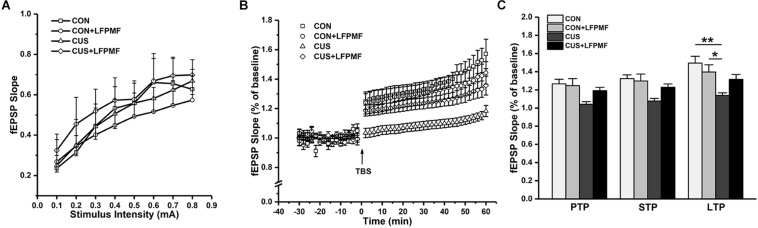
Basal synaptic transmission and synaptic plasticity recorded from Schaffer collaterals to CA1 region. **(A)** I/O curves: slopes of EPSPs were plotted against stimuli ranging from 0.1 to 0.8 mA. **(B)** The time coursing of normalized EPSPs slopes in LTP stage. **(C)** Histogram shows the average changes in EPSPs slopes in PTP, STP, and LTP. ^∗^*p* < 0.05, ^∗∗^*p* < 0.01.

In the electrophysiological experiment, the induction of LTP did not succeed in all rats. There were CON (*n* = 4), CON + LFPMF (*n* = 4), CUS (*n* = 7), CUS + LFPMF (*n* = 4). EPSPs were evoked by a range of stimuli from Schaffer collaterals to CA1. To quantify these responses, measurements were taken from the slope of EPSPs. The slopes of I/O curves from Schaffer to CA1 in CON + LFPMF, CUS or CUS + LFPMF were similar to CON rats [Figure 4A, main effect of time: *F*(7, 98) = 82.655, *p* < 0.001, main effect of treatment: *F*(1, 14) = 0.004, *p* = 0.950, main effect of CUS model: *F*(1, 14) = 0.347, *p* = 0.565, no CUS model × treatment interaction: *F*(1,14) = 0.429, *p* = 0.523, repeated measures ANOVA]. After TBS, LTP in CON + LFPMF and CUS + LFPMF rats was sustained at the same level as CON rats for the duration of the recordings [Figure 4B, main effect of time: *F*(29, 406) = 50.596, *p* < 0.001, main effect of treatment: *F*(1, 14) = 1.414, *p* = 0.254, repeated measures ANOVA]. In contrast, potentiation significantly declined in CUS model rats by 60 min after LTP induction [Figure 4B, main effect of CUS model: *F*(1, 14) = 13.005, *p* = 0.003, no CUS model × treatment interaction: *F*(1,14) = 4.527, *p* = 0.052, repeated measures ANOVA]. The synapses also showed two forms of brief potentiation: post-tetanic potentiation (PTP), which lasts for 5–10 min, and short-term potentiation (STP), which lasted rather longer, around 30 min (Hannay et al., 1993). Following the brief potentiation, the LTP was generated and lasted for an hour or longer (Colino et al., 1992). Therefore, the slopes of different stages were analyzed to evaluate the differences among the four groups. As shown in Figure 4C, there were significant differences in the three phases [PTP: main effect of treatment: *F*(1, 14) = 1.778, *p* = 0.204, main effect of CUS model: *F*(1, 14) = 8.176, *p* = 0.013, no CUS model × treatment interaction: *F*(1,14) = 2.947, *p* = 0.108; STP: main effect of treatment: *F*(1, 14) = 1.856, *p* = 0.195, main effect of CUS model: *F*(1, 14) = 11.575, *p* = 0.004, no CUS model × treatment interaction: *F*(1,14) = 3.638, *p* = 0.077; LTP: main effect of treatment: *F*(1, 14) = 0.460, *p* = 0.509, main effect of CUS model: *F*(1, 14) = 14.245, *p* = 0.002, CUS model × treatment interaction: *F*(1,14) = 5.715, *p* = 0.031, two-way ANOVA].

### The Effects of LFPMF on Neural Oscillation in the Hippocampus

**FIGURE 5:**
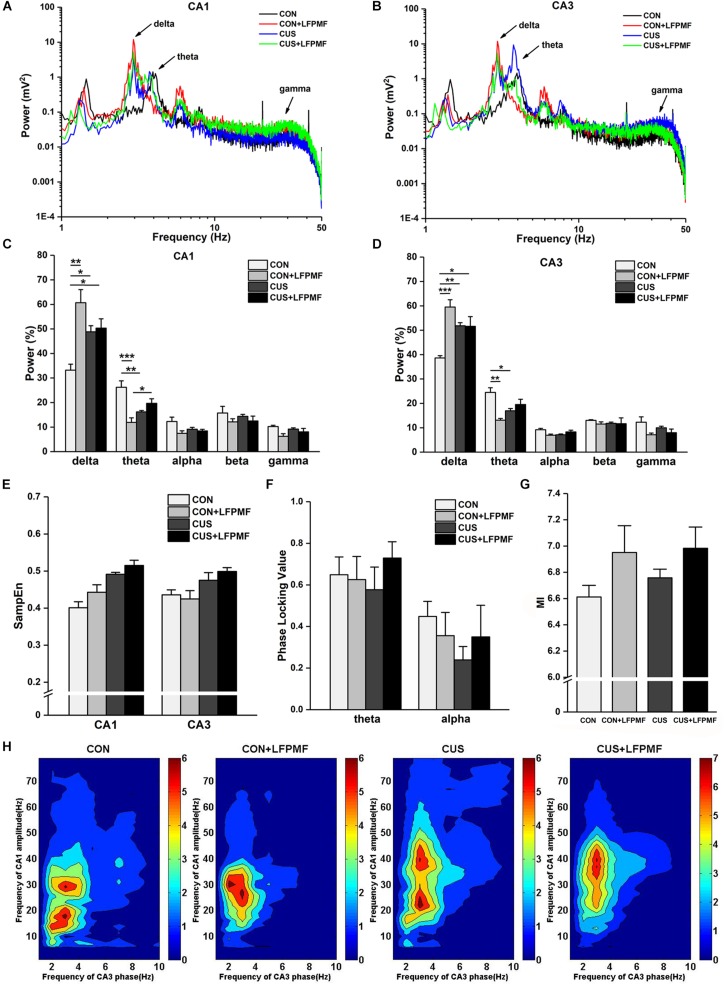
Changes of power within various frequency bands and neural oscillation in hippocampus. **(A,B)** Representative traces of CA1 and CA3 LFPs in CON, CON + LFPMF, CUS, and CUS + LFPMF groups. **(C)** The group data of the power of various rhythms in both CA1 and **(D)** CA3 regions. **(E)** Non-linear sample entropy (SampEn) of LFP in CA1 and CA3 regions. **(F)** Averaged Schaffer-CA1 PLV at the theta rhythm and alpha rhythm. **(G)** Statistical MI data of phase-amplitude coupling between theta and gamma rhythms in the hippocampal Schaffer-CA1 pathway. **(H)** Mean PAC-MI measurement (Z-scored) of hippocampus in four groups. ^∗^*p* < 0.05, ^∗∗^*p* < 0.01, ^∗∗∗^*p* < 0.001.

Signals of LFP in Schaffer collaterals and CA1 were collected simultaneously. Represented LFP power spectra are shown in Figures 5A,B. The grand average is illustrated in Figures 5C,D, which includes the data of CA1 and CA3 as well as various frequency bands containing delta (1–4 Hz), theta (4–8 Hz), alpha (8–15 Hz), beta (15–30 Hz), and gamma (30–50 Hz). There were significant differences of power density at delta and theta rhythms both in CA1 [Figure 5C, delta rhythm, main effect of treatment: *F*(1, 14) = 16.733, *p* = 0.001, main effect of CUS model: *F*(1, 14) = 0.553, *p* = 0.469, CUS model × treatment interaction: *F*(1,14) = 13.426, *p* = 0.003; theta rhythm, main effect of treatment: *F*(1, 14) = 10.143, *p* = 0.007, main effect of CUS model: *F*(1, 14) = 0.435, *p* = 0.520, CUS model × treatment interaction: *F*(1,14) = 27.414, *p* < 0.001, two-way ANOVA] and CA3 regions [Figure 5D, main effect of treatment: *F*(1, 14) = 18.105, *p* = 0.001, main effect of CUS model: *F*(1, 14) = 1.192, *p* = 0.293, CUS model × treatment interaction: *F*(1,14) = 18.912, *p* = 0.001; theta rhythm, main effect of treatment: *F*(1, 14) = 9.181, *p* = 0.009, main effect of CUS model: *F*(1, 14) = 0.145, *p* = 0.709, CUS model × treatment interaction: *F*(1,14) = 22.760, *p* < 0.001, two-way ANOVA]. The pattern of field neural activity in depression rats was slightly changed by LFPMF.

Sample entropy (SampEn) of LFP is presented in Figure 5E. The analysis demonstrated that SampEn values were not significantly changed among the four groups in either CA1 [main effect of treatment: *F*(1, 14) = 0.952, *p* = 0.346, main effect of CUS model: *F*(1, 14) = 1.842, *p* = 0.196, no CUS model × treatment interaction: *F*(1,14) = 0.170, *p* = 0.687, two-way ANOVA] or CA3 [main effect of treatment: *F*(1, 14) = 1.019, *p* = 0.330, main effect of CUS model: *F*(1, 14) = 0.481, *p* = 0.499, no CUS model × treatment interaction: *F*(1,14) = 0.047, *p* = 0.831, two-way ANOVA]. However, both in CA1 and CA3 regions, the SampEn of model groups was slightly higher than control groups.

Although the differences were not significant, a trend of decrease occurred in the CUS group of the PLV on theta [Figure 5F, main effect of treatment: *F*(1, 14) = 0.385, *p* = 0.545, main effect of CUS model: *F*(1, 14) = 0.024, *p* = 0.880, no CUS model × treatment interaction: *F*(1,14) = 0.708, *p* = 0.414, two-way ANOVA] and alpha [main effect of treatment: *F*(1, 14) = 0.007, *p* = 0.934, main effect of CUS model: *F*(1, 14) = 1.115, *p* = 0.309, no CUS model × treatment interaction: *F*(1,14) = 0.996, *p* = 0.335, two-way ANOVA] rhythms. Additionally, it can be seen that there was a visible theta-gamma PAC in CON and LFPMF groups [Figure 5G, main effect of treatment: *F*(1, 14) = 4.664, *p* = 0.049, main effect of CUS model: *F*(1, 14) = 0.460, *p* = 0.508, no CUS model × treatment interaction: *F*(1,14) = 0.192, *p* = 0.668, two-way ANOVA]. Additionally, Figure 5H shows the mean modulation indices in all groups.

### Effects of LFPMF on the Expression of Related Synaptic Proteins

**FIGURE 6:**
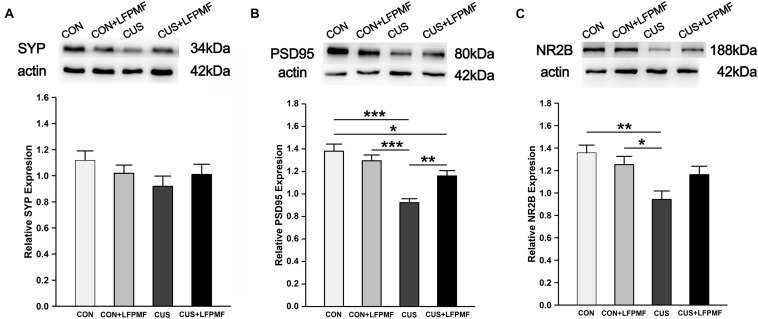
Effects of LFPMF on the expression of synaptic-related proteins in hippocampus. Representative immunoreactive bands of SYP **(A)**, PSD95 **(B)**, NR2B **(C)**, and β-actin (upper) and quantitative analysis of the optical density ratio of SYP/β-actin **(A)**, PSD95/β-actin **(B)**, and NMDAR2B/β-actin **(C)**. ^∗^*p* < 0.05, ^∗∗^*p* < 0.01, ^∗∗∗^*p* < 0.001.

Our data so far demonstrated that CUS disrupted the LTP of the Schaffer-CA1 pathway. Synaptic and extrasynaptic N-methyl-D-aspartic acid receptor (NMDAR) proteins play different roles in regulating synaptic plasticity. We therefore measured the levels of synaptic-related proteins including synaptophysin (SYP), postsynaptic density protein 95 (PSD95), and NMDAR 2B (NR2B). The protein content of SYP (∼43 kDa) in the brain of CUS rats was lower than other rats [Figure 6A, main effect of treatment: *F*(1, 120) = 0.003, *p* = 0.958, main effect of CUS model: *F*(1, 120) = 2.007, *p* = 0.159, no CUS model × treatment interaction: *F*(1,120) = 1.671, *p* = 0.199, two-way ANOVA], although the differences were not significant. Significant differences were present in the relative expression of PSD95 (∼80 kDa) [Figure 6B, main effect of treatment: *F*(1, 132) = 2.255, *p* = 0.136, main effect of CUS model: *F*(1, 132) = 34.865, *p* < 0.001, CUS model × treatment interaction: *F*(1,132) = 10.312, *p* = 0.002, two-way ANOVA] and NR2B (∼166 kDa) [Figure 6C, main effect of treatment: *F*(1, 80) = 0.623, *p* = 0.432, main effect of CUS model: *F*(1, 80) = 11.907, *p* = 0.001, CUS model × treatment interaction: *F*(1,80) = 4.989, *p* = 0.028, two-way ANOVA]. *Post hoc* analysis revealed that LFPMF in the CUS rats caused a significant increase in hippocampal PSD95 and NR2B levels, while LFPMF had no distinct effect on CON rats.

## Discussion

Transcranial magnetic stimulation was introduced as a diagnostic method in 1985 (Barker et al., 1985), and recently it has been well established in neurology. Given that stimulation in different parameters leads to different outcomes, here, in the present study, we demonstrated that LFPMF (1 Hz, 20 mT) relieved the impaired spatial cognition induced by CUS. The potential mechanisms were correlated with the alternation of hippocampal synaptic efficacy and neural oscillation. These results indicated that a feasible and effective TMS protocol at low frequency has the potential to treat cognitive impairment in depression.

Chronic unpredictable stress as a well-validated paradigm has been used to induce a depression-like syndrome with some degree of cognitive deficit (Bondi et al., 2008; Logan et al., 2015), which was selected in this study. Compared to control groups, the CUS model animals showed typical depression symptoms in a SPT. It is noticeable, however, compared with the CON group, that the other three groups had anxiety symptoms observed in EPM. Nevertheless, in EPM the differences of the percentages of residence time in open arms among groups were not significant. This may be due to the sample size and differences within groups. As expected, the stress rats (CUS treated) also showed abnormal memory function which have been shown in MWM.

In order to assess the protective effect of LFPMF on cognition, MWM was set out to perform the training and probe trials. In the training trial, spatial learning is assessed across repeated trials, while in the probe trial reference memory was determined by preference for the platform area when the platform was absent. The reversal phase can reveal whether animals can extinguish their initial learning and memory of the platform’s position and acquire a direct path to the new goal position. This method was used to evaluate the flexibility of learning and self-regulation (Vorhees and Williams, 2005). Most people with depression cannot regulate emotions very well, thus they are likely to be sustained in negative emotion (Ehring et al., 2010; Joormann and Vanderlind, 2014). Animal studies found that the impairment of reversal learning and behavioral flexibility was related to the disruption of LTD (Mills et al., 2014). Furthermore, reversal learning has been shown to involve disparate brain functional connectivity, including prefrontal connectivity and functional connectivity between the hippocampus and cingulate cortex (de Bruin et al., 1994; Shah et al., 2018). In this study, the results revealed that LFPMF could improve the spatial memory ability of rats, while they reacted quickly to the change of the platform’s position. Therefore, our results implied that LFPMF as an effective and non-invasive brain stimulation method might improve cognition impairments induced by CUS.

The spatial cognition is strongly correlated with hippocampal synaptic plasticity and synaptic proteins. Furthermore, there is mounting evidence that damage to the hippocampus can produce inflexible and maladaptive behavior in humans (Henke et al., 1999; Mumby et al., 2002; Rubin et al., 2014) in areas such as memory, navigation, exploration, establishing and maintaining social bonds, etc. In different hippocampal subfields, the CA1 region is crucial for behavioral cognition, especially for spatial cognition, while the CA3 region is important for memory retention (Noble et al., 2014; Shang et al., 2016). The synaptic connection from CA3 Schaffer collaterals to CA1 pyramidal neurons belongs to the trisynaptic circuit of the hippocampus, which plays an important role in learning and memorizing (Scullin and Partridge, 2012). Hence, we measured multiform synaptic plasticity of the CA3-CA1 pathway, including long-term and short-term plasticity (Ohno et al., 2011). As mentioned before, LTP could last for an hour or longer (Colino et al., 1992). In our control group, the slope of fEPSPs was higher over time after TBS, which is consistent with some published articles (Petit-Pedrol et al., 2018; Wang et al., 2019). Previous studies have shown that depression impairs LTP, which may be a direct manifestation of abnormal neural plasticity (Shang et al., 2016; Wang et al., 2017b; Yang et al., 2017). In particular, the idea that LTP in the hippocampus supports associative memory formation has been widely accepted and has only rarely been questioned (Bannerman et al., 2014). As for short-term plasticity, it has been experimentally characterized in hippocampal neurons and considered to be one candidate mechanism for short-term memory (Erickson et al., 2010; Wang et al., 2017b; Tian et al., 2018). In the present study, both long-term and short-term plasticity of the CA3-CA1 pathway in CUS was ameliorated by LFPMF, in line with behavioral manifestations of MWM. As is widely known, hippocampal synaptic proteins are critical for synaptic plasticity (Fox et al., 2010; Counts et al., 2014; Shang et al., 2016; Han et al., 2018). It has been found that NMDAR plays an important role in the induction of LTP (Barkus et al., 2010). Thus, as one of the NMDAR subunits, NR2B is also believed to be closely related to synaptic plasticity and cognitive function (Barkus et al., 2010; Fox et al., 2010). There are other synaptic proteins which are also related to synaptic function, such as SYP and PSD95. The former is an essential membrane protein in synaptic vesicle, while PSD95 protein is present in the core part of the postsynapse (Counts et al., 2014; Shang et al., 2016; Han et al., 2018). In this study, the semiquantitative changes (Figure 6) implied that the damage of CUS in depression may be mainly relevant to a postsynaptic mechanism rather than presynaptic. Crucially, after LFPMF, the damage could be alleviated, which implied that the target of LFPMF may be postsynaptic specific. In short, the up-regulation of synaptic plasticity and postsynaptic protein expression in the hippocampus may be the mechanisms of LFPMF on depression.

Apart from synaptic function, neural oscillation is also closely associated with cognition. The central problem for cognitive neuroscience is to describe how cognitive processes arise from brain processes. Informed by modern functional imaging techniques such as PET and fMRI, previous research has made an impressive beginning on this task. But cognitive processes are not static; they are dynamic (Ward, 2003). Rhythms with different frequencies in neural oscillation are related to various brain processes. Memory processes are most closely related to theta and gamma rhythms (Ward, 2003; Xu et al., 2015, 2016; Zheng and Zhang, 2015). Although the pathway from the ventral hippocampus to the medial prefrontal cortex (mPFC) is thought to play a significant role in emotional memory processing, the hippocampal CA3-CA1 pathway is recognized to be closely linked with spatial cognition (Montgomery and Buzsáki, 2007; Li et al., 2013; Wu et al., 2013; Kojima et al., 2016). Thus, neural oscillation of the CA1 and CA3 regions in the hippocampus was analyzed in this study. Moreover, the previous study (Wang et al., 2011) found that LFPMF (1 Hz) could increase the energy of low frequency bands (1–40 Hz) of human brain, especially the band of 1–3 Hz, which is called “resonance effects.” Our animal experiments were consistent with the previous findings. LFPMF had a strong effect in CON rats, especially on delta and theta activities. The power of delta rhythm in CON rats was significantly lower than other groups, and we speculate that this could be induced by CUS treatment and 1 Hz magnetic stimulation, which could exert an effect on neuronal rhythmic activity of rats, especially on low-frequency rhythm. In some investigations, delta activity was significantly increased in animals of the Katz model, a depression model similar to CUS (Sarbadhikari et al., 1996). In our previous research, furthermore, it was found that LFPMF significantly improved the undesirable changes of the identical-frequency synchronization and theta-gamma phase-amplitude coupling in CUS rats’ hippocampi (Wang et al., 2018). With a few exceptions, in this paper LFPMF made an increasing tendency in the phase synchronization and the phase-amplitude coupling of the CUS group, but there was no significant difference. The cause may be that the number of animals used in neural oscillation analysis was not enough. On the other hand, there may also be other parameters that are affected which were not measured. In short, these results suggest that the regulation of neural oscillation in the hippocampus may be the potential mechanism of LFPMF, which needs further research in the future.

So far, we have tried to reveal the potential mechanisms of LFPMF from two aspects, synaptic function and neural oscillation, which have been proved to be closely related. For example, it has been found that theta phase coupling was positively correlated with synaptic plasticity in the vCA1–mPFC pathway in depression rats (Zheng and Zhang, 2015). In addition, Xu et al. (2013) reported that CA3-CA1 synaptic plasticity was positively correlated with the unidirectional indices from CA3 to CA1 in melamine-treated rats. This study showed that low-frequency magnetic stimulation can modulate synaptic function of CUS-induced depressive rats. The parameters we used in neural oscillation presented the effect of LFPMF in control rats rather than CUS model rats. Thus, other methods of neural oscillation analysis need further investigation, as well as the relationship between synaptic function and neural oscillation.

Overall, our study demonstrated that LFPMF could ameliorate the deficits of cognitive and synaptic functions through up-regulating the expression of related proteins in CUS rats, suggesting that LFPMF may serve as an effective treatment of cognition impairment caused by depression or other diseases. In the future, the molecular mechanisms underlying the beneficial effects of LFPMF and its remarkable effects on mood need to be further investigated.

## Data Availability

Publicly available datasets were analyzed in this study. This data can be found here: https://doi.org/10.5281/zenodo.3261843.

## Ethics Statement

This study was carried out in accordance with the recommendations of Animal Management Rules of the Ministry of Health of the People’s Republic of China. The protocol was approved by the Animal Research Ethics Committee, School of Medicine, Nankai University.

## Author Contributions

JY and DM conceived and designed the manuscript. JY and LW drafted the manuscript. LW, FW, and RL performed the behavioral and electrophysiological experiments. LW and XT performed the western blot assay. PZ provided the technical support on magnetic stimulation. CZ contributed to the data analysis. All authors read and approved the final version of the manuscript.

## Conflict of Interest Statement

The authors declare that the research was conducted in the absence of any commercial or financial relationships that could be construed as a potential conflict of interest.
